# *YPK9* and *WHI2* Negatively Interact during Oxidative Stress

**DOI:** 10.3390/microorganisms9122584

**Published:** 2021-12-14

**Authors:** Florenal Joseph, Darach Miller, Oleg V. Evgrafov, William J. Chirico

**Affiliations:** 1 Molecular and Cellular Biology Program, School of Graduate Studies, SUNY Downstate Health Sciences University, New York, NY 11203, USA; florenal.joseph@nyulangone.org; 2 Joint Initiative for Metrology in Biology, SLAC National Accelerator Laboratory, Menlo Park, CA 94025, USA; darachm@stanford.edu; 3 Department of Genetics, Stanford University, Stanford, CA 94305, USA; 4 Department of Cell Biology, SUNY Downstate Health Sciences University, New York, NY 11203, USA; oleg.evgrafov@downstate.edu

**Keywords:** yeast, *Saccharomyces cerevisiae*, hydrogen peroxide, ATP13A2, Parkinson’s disease

## Abstract

Yeast *PARK9* (*YPK9*) shares homology with human *ATP13A2*, which encodes a polyamine transporter implicated in juvenile forms of Parkinson’s disease. We used *YPK9* to gain insight into how *ATP13A2* affects cell growth and sensitivity to oxidative stress. Surprisingly, the *YPK9* deletion strain from the *Saccharomyces cerevisiae* deletion collection (YKO) in wildtype BY4741 (mating type a) grew faster and was more resistant to hydrogen peroxide than a commercial, putative parental BY4741 wildtype strain (BY4741^COM^). In contrast, deleting *YPK9* from BY4741^COM^ rendered it very sensitive to hydrogen peroxide, suggesting its background is different from that of the deletion collection. Whole-genome sequencing revealed that BY4741^COM^ and BY4741^COM^ *ypk9*∆ contain a novel premature stop codon near the 3′ end of *WHI2* (*WHI2*^G1324T^), whereas the collection’s *YPK9* deletion strain contains *WHI2*, which encodes a 486 amino acid protein, Whi2p. Replacing full-length *WHI2* with the sequence coding for the predicted truncation (Whi2p^E442*^) rendered strains more sensitive to hydrogen peroxide, whereas the converse replacement rendered them more resistant. The sequences of *WHI2* in 20 randomly chosen strains from the collection encode the full-length protein, indicating that the putative parental BY4741 *WHI2*^G1324T^ strain’s genetic background differs from that of the deletion collection. Examination of *WHI2* sequences in several commonly used wildtype *S. cerevisiae* strains and isolates revealed other Whi2p truncations that might yield altered phenotypes. Together, these results demonstrate a novel premature stop codon in *WHI2* that renders yeast sensitive to hydrogen peroxide; they also reveal a negative genetic interaction between *WHI2* and *YPK9* in the presence of hydrogen peroxide in the BY4741 background.

## 1. Introduction

Lindquist et al. first identified *YPK9* as a suppressor of α-synuclein toxicity in yeast [1]. α-synuclein is the major component of Lewy bodies, which are cytoplasmic structures that are the hallmark of Parkinson’s disease. *S. cerevisiae YPK9* is 38% identical to human *ATP13A2* [2], which encodes a multispanning membrane protein of lysosomes that exports polyamines to the cytosol [3]. Mutations in human *ATP13A2* are associated with Kufor–Rakeb syndrome, an early onset form of Parkinson’s disease [4]. Polyamines are organic polycations with diverse functions including binding nucleic acids (RNA and DNA), scavenging reactive oxygen species (ROS), and activating eIF5A (yeast Hyp2p) by hypusination, an essential post-translational modification [5,6]. Thus, the convergence of oxidative stress, protein condensates, and polyamine metabolism on *YPK9* may yield insight into the dysregulation of fundamental pathways underlying Parkinson’s disease.

*WHI2* was originally identified by Sudbery et al. [7] during a screen for cell-cycle mutants. The *whi2* mutant yielded small, mostly budding cells in the stationary phase. In 2008, Hardwick et al. showed that cells lacking *FIS1*, a gene required for mitochondrial fission, acquired a secondary mutation, a premature stop codon in *WHI2*, rendering it presumably inactive and allowing the cells to grow in amino-acid-deficient media, in particular leucine-deficient media [8]. *WHI2*, in essence, acts like a leucine sensor. *WHI2* was shown to block TORC1, a complex of proteins that regulates cell metabolism and protein translation in yeast and higher eukaryotes [9]. Thus, under limiting leucine levels, cell survival is enhanced by *WHI2* blocking TORC1, thereby attenuating translation and increasing autophagy. These findings extend those of Costanzo et al. [10], who showed in a large-scale screen that *WHI2* interacts with certain members of TORC1. Thus, identifying and characterizing novel *whi2* mutants may deepen our understanding of core conserved eukaryotic growth signaling pathways.

During our recent investigation into the role of *YPK9* in peroxisomal proliferation, we noticed that two *YPK9* deletion strains in BY4741 had different sensitivities to hydrogen peroxide: a laboratory-generated deletion was much more sensitive than *ypk9*∆ from the yeast deletion collection. In the studies described below, we used whole-genome sequencing to identify *WHI2* as the gene responsible for the difference in oxidative stress sensitivity and revealed its negative genetic interaction with *YPK9*.

## 2. Materials and Methods

### 2.1. Yeast Strains, Media, and Plasmid

The strains used in this study are described in Table S1. Strains were cultured at 30 °C in YPD (1% yeast extract, 2% peptone, and 2% glucose) or synthetic complete media (CSM (Sunrise Science, Knoxville, TN, USA) supplemented with yeast nitrogen base (YNB) and 2% glucose). For growth on agar plates, YPD medium was supplemented with 2% agar. The yeast deletion collection was obtained from Thermo Scientific (YKO-MATa, #YSC1053). Hygromycin (Hyg), nourseothricin (NTC), and G418 were obtained from Gold Biotechnology and used at 100, 100, and 200 µg/mL, respectively. Hydrogen peroxide (30%) was purchased from Fisher Scientific. pAG32 was a gift from John McCusker (plasmid #35122; Addgene, Watertown, MA, USA).

### 2.2. Strain Genome Sequencing and Analysis

We used a DropSense 96 instrument (PerkinElmer, Waltham, MA, USA) and DropQuant software to quantify and assess the purity of input DNA. We performed whole-genome library preparation according to the on-bead tagmentation protocol (Illumina doc# 1000000025416 v06) using 500 ng of input DNA for each sample. Libraries were sequenced on a NovaSeq 6000 sequencer (Illumina) as 2 × 150 reads with an average number of reads of 16,513,549 (4954 Mb) per sample. Sequencing reads were aligned to the *S. cerevisiae* reference genome (SacCer3, [11]) using Burrows-Wheeler Aligner (BWA, [12]). Single nucleotide polymorphisms (SNPs) and insertion-deletion (INDEL) variants were visualized with the standalone Integrative Genomics Viewer (IGV) browser (Broad Institute, Cambridge, MA, USA).

### 2.3. Construction of WHI2 Variants

We used the method of gene replacement to introduce *WHI2* or *WHI2*^G1324T^ in various strains [13]. In the first step, a hygromycin cassette was amplified from pAG32 [14]. The forward primer contained 45 bases of the 3′ terminus of *WHI2* and 22 bases of the cassette (WHI2&HYG_fwd: CGTAGAGTTTGGACTTTAGAGTTGAGCGTTATTGGGGTGCAGTGACAGCTGAAGCTTCGTACGCTGC). The reverse primer contained 45 bases immediately downstream of *WHI2* and 22 bases of the cassette (WHI2&HYG_rev) TGGCCCGATCTCTTTCCATTTCTTTCTCTAATATATTATATACACGCATAGGCCACTAGTGGATCTG). The amplified product was used to transform BY4741^COM^, which contained *WHI2*^G1324T^ and BY4742, which contained *WHI2*. The *WHI2*^G1324T^-HYG and *WHI2*-HYG fusions were amplified from genomic DNA isolated from the transformants. Genomic DNA was extracted as previously described [15]. The forward primer (WHI2_UP_45) was GATAAAGATAAAGGTTGTCTGAGCTTACACTTATTATAAACAATG and the reverse primer (WHI2_DN_42) was CCCGATCTCTTTCCATTTCTTTCTCTAATATATTATATACAC [16]. The amplified products were used to exchange *WHI2*^G1324T^ and *WHI2* in certain strains. Phusion High-Fidelity DNA Polymerase (New England Biolabs, Ipswich, MA, USA) and Buffer GC were used in amplification reactions following the manufacturer’s instructions. The sequence of mutant and wildtype *WHI2* in all strains was verified by DNA sequencing. The forward sequencing primer (WHI2_FWD_710p) was AGACTAAAGCAACAACAGCAAC. The reverse primer (WHI2-SEQ-R-REV: GGGATACCAAGAAACCATACTG) was used to verify the *WHI2* sequence in randomly selected strains from the yeast deletion collection.

### 2.4. Yeast Growth Assays

Frozen stocks of strains were used to prepare fresh streaks on YPD agar plates, samples of which were used to inoculate 5 mL of YPD with or without antibiotics, and the cultures were rotated overnight at 30 °C. Cultures were diluted to 0.05 OD_600_ and three 200 μL aliquots of each strain were placed in a sterile 96-well microplate. For background correction, certain wells contained only medium. Cultures were incubated in a Sunrise Tecan microplate reader at 25 °C for 2 h with shaking. Hydrogen peroxide (50 µM final concentration) or water was added to the cultures and the incubation was continued for a total of 24 h. The OD_600_ was recorded in 15 min intervals.

### 2.5. Calculation of the Strength of Genetic Interactions

The strength of a genetic interaction (ε) was calculated using a multiplicative model [17]. The following equation was used:

(1) $$\varepsilon =f_{ab}-f_{a}f_{b}$$

where f*_a_* and f*_b_* are the fitness of single mutants *a* and *b*, respectively; and f*_ab_* is the observed fitness of the double mutant. Fitness was defined as the OD_600_ after 24 h of growth in the presence of hydrogen peroxide normalized to the wildtype. A positive ε signifies a positive interaction, whereas a negative ε signifies a negative interaction.

## 3. Results

**Figure 1:**
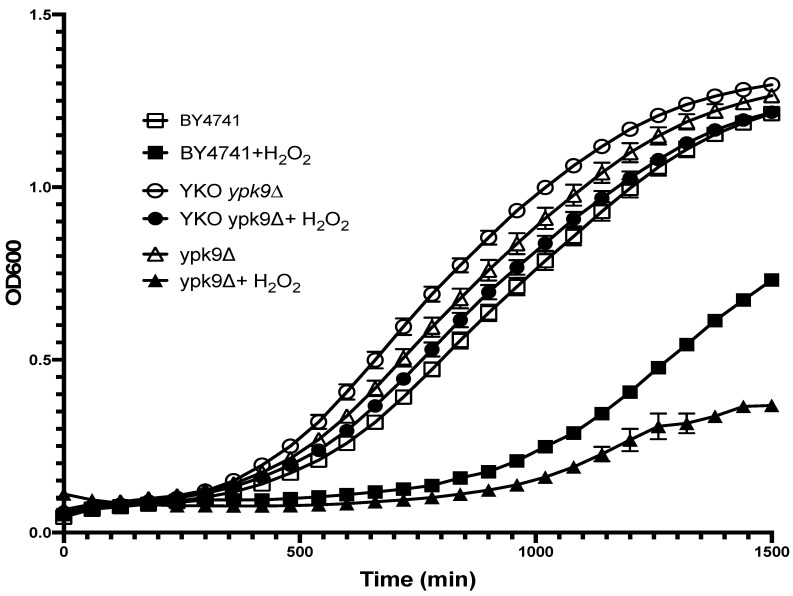
H_2_O_2_ sensitivity of the YKO collection and laboratory *YPK9* deletion strains. The growth curves of BY4741^COM^ (squares), the YKO collection’s *ypk9*∆ in BY4741 (circles), and our *ypk9*∆ in BY4741^COM^ (triangles) were compared in the presence (solid symbols) or absence (open symbols) of 50 µM H_2_O_2_. Differences in growth (see text) were based on OD_600_ at mid-log point. Error bars represent the SEM, *N* = 3.

Based on *YPK9*’s sequence homology to human *ATP13A2*, we speculated that a strain lacking *YPK9* would have a growth defect and be sensitive to oxidative stress. We found, however, that the YKO collection’s *ypk9*∆ strain grew slightly better (~15%, based on OD_600_ at mid-log point) than the commercial strain putatively isogenic to the parental strain, BY4741^COM^ (Figure 1). Because oxidative damage accumulates during ageing, we sought to uncover a sensitivity to oxidative stress by treating YKO’s *ypk9*∆ with hydrogen peroxide. Hydrogen peroxide reduced the mid-log phase growth of YKO’s *ypk9*∆ by only 19%, but that of BY4741^COM^ by 32% relative to the corresponding untreated strains, suggesting that loss of *YPK9* rendered BY4741 more resistant to oxidative stress (Figure 1). To corroborate this unexpected result, we deleted *YPK9* from BY4741^COM^ and measured its growth and hydrogen peroxide sensitivity. The growth of this BY4741^COM^ *ypk9*∆ deletion strain at mid-log phase was about 14% less than that of the YKO *ypk9*∆ strain (Figure 1). In the presence of hydrogen peroxide, however, the BY4741^COM^ *ypk9*∆’s growth in the mid-log phase was severely hampered: about 68% less than YKO *ypk9*∆’s. At least two explanations are possible for the difference in hydrogen peroxide sensitivity of the two *ypk9*∆ strains. First, the YKO *ypk9*∆ strain may have acquired a suppressor of oxidative stress since the collection was constructed [16]. Hardwick et al. estimated that the majority of strains in the YKO collection have acquired a suppressor [18]. Second, the commercial, putative parental BY4741^COM^ may not share the same genetic background as the deletion collection.

To identify genomic differences that may explain these results, we sequenced the genomes of BY4741^COM^, BY4742, YKO *ypk9*∆, and BY4741^COM^ *ypk9*∆. The four strains had a total of 290 sequence changes relative to the SacCer3 reference genome (Table S2). Of those, 202 were common to all four strains and could not provide insight into the phenotypic differences among them. Most of the remaining variants (88) were short single-nucleotide insertions, deletions, or SNPs located between ORFs and, therefore, of uncertain significance. However, we detected variants in four genes (*ADH7*, *DNF2*, *RAX1,* and *WHI2*) that were shared between BY4741^COM^ and our *ypk9*∆ BY4741^COM^ strain but not with the YKO *ypk9*∆ (Table 1).

Alcohol dehydrogenase 7 (*ADH7*) is an NADP-dependent alcohol dehydrogenase and member of the cinnamyl alcohol dehydrogenase family [19]. We identified a G-to-A SNP at position 309,566 in chr III predicted to change Gly-166 to Asp. This sequence change was characterized as a passenger mutation and is therefore unlikely to affect growth [20]. *DNF2* is an aminophospholipid translocase (flippase) [21,22]. We identified a G-to-C SNP at position 632,116 on chr IV predicted to change Gly-279 to Arg. This SNP in *DNF2* was not found among the gene variants listed on the *Saccharomyces* Genome Database (SGD [23]). *RAX1* is involved in bud site selection [24,25]. We identified a C-to-A SNP at position 881,528 predicted to change a Cys-188 to a stop codon. The full-length protein has 435 amino acid residues. *WHI2* is a negative regulator of TORC1 and is required for the activation of the general stress response in yeast. We identified a G-to-T SNP at position 412,193 in chr XV predicted to change a Glu-442 codon to a stop codon yielding Whi2p^E442*^. Full-length Whi2p has 486 amino acid residues. Neither the *RAX1* nor *WHI2* variant described here was found in the SGD [23].

**Figure 2:**
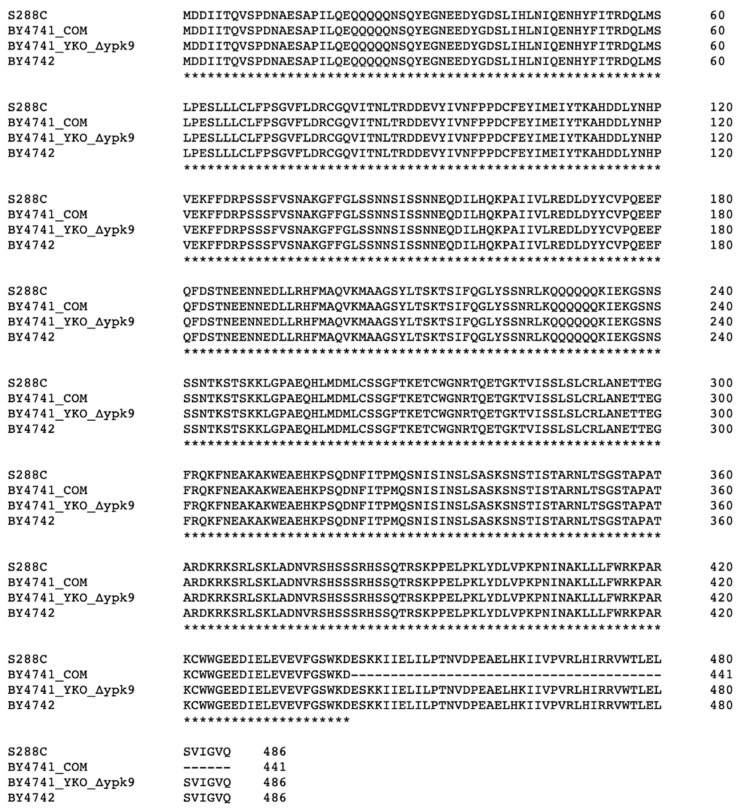
Alignment of Whi2p variants. The reference strain (S288C), BY4742, and YKO collection’s *ypk9*∆ contain the sequence for full-length Whi2p (486 amino acids). In contrast, BY4741^COM^’s *WHI2* sequence has a premature stop codon yielding a truncated Whi2p of 441 amino acids residues. Clustal Omega was used to align sequences [29].

We focused our attention on *WHI2* because it plays a key role in the general stress response [26,27,28], regulates TORC1 [9], and is one of five genes most commonly mutated in strains containing a suppressor [20]. Full-length *WHI2* is found in YKO’s BY4741 *ypk9*∆ and BY4742, whereas *WHI2*^G1324T^ is found in BY4741^COM^ and BY4741^COM^ *ypk9*∆ (Figure 2).

**Figure 3:**
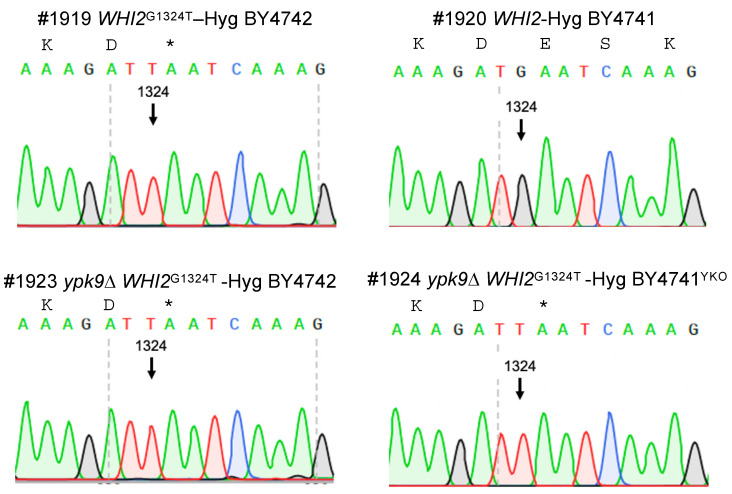
Sequence confirmation of engineered *WHI2* variants. A hygromycin cassette was fused to *WHI2* or its variant containing a stop codon at position 1324 (*WHI2*^G1324T^) and used to replace the endogenous version of *WHI2* in BY4741^COM^, BY4742, and YKO collection’s *ypk9*∆ in BY4741^YKO^. DNA sequencing was used to confirm the replacement. The asterisk represents the stop codon. Numbers with a hashtag represent the laboratory strain identification number.

**Figure 4:**
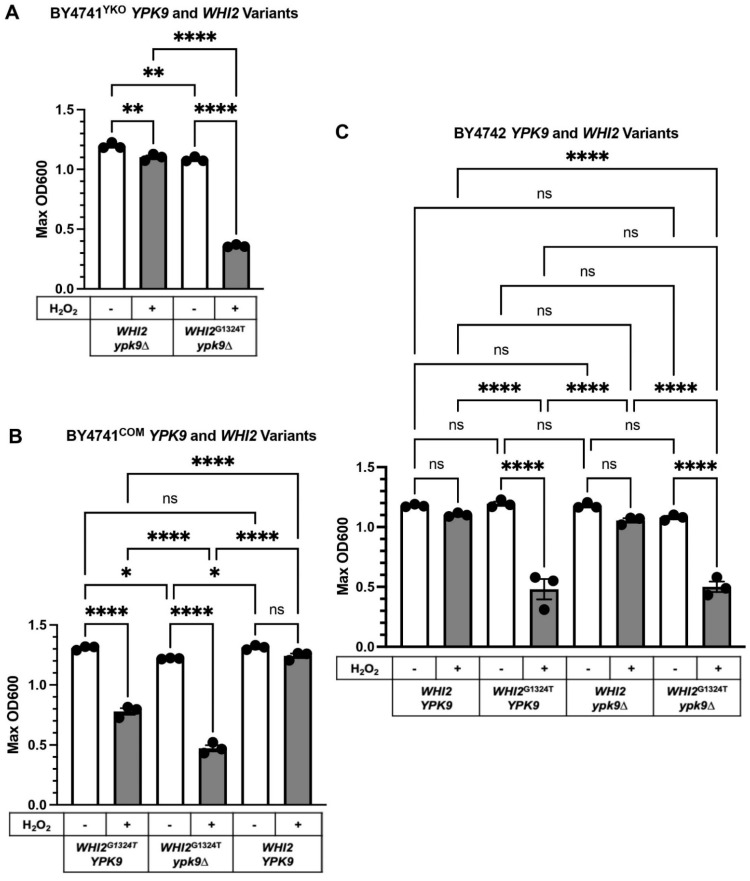
Effect H_2_O_2_ on the growth of *WHI2* and *YPK9* variants in different background strains. (**A**) *WHI2* variants in the YKO collection’s *ypk9*∆ in BY4741^YKO^. (**B**) *WHI2* variants in BY4741^COM^. (**C**) *WHI2* and *YPK9* variants in BY4742. Hygromycin (Hyg) was used to select for gene replacement. The OD shown is the maximum recorded during 25 h of growth. Results were analyzed using one-way ANOVA and Tukey’s post hoc test. Error bars represent the SEM, *n* = 3. Asterisks represent *p*-values: * 0.03332, ** 0.0021, **** < 0.0001; ns, not significant.

We tested the hypothesis that *WHI2*^G1324T^ renders BY4741^COM^ and BY4741^COM^ *ypk9*∆ sensitive to hydrogen peroxide by systematically exchanging *WHI2* and *WHI2*^G1324T^ in our strains (Figure 3) and then measuring their sensitivity to hydrogen peroxide (Figure 4). Hydrogen peroxide reduced the growth of BY4741^YKO^ *ypk9*∆ by about 8% compared to its untreated control (Figure 4A). Replacing *WHI2* with *WHI2*^G1324T^ in *ypk9*∆ BY4741^YKO^ rendered it very sensitive to hydrogen peroxide reducing growth by 67% (Figure 4A).

We extended the analysis by replacing *WHI2*^G1324T^ with *WHI2* in BY4741^COM^ and comparing its hydrogen peroxide sensitivity to that of BY4741^COM^ *WHI2*^G1324T^. As expected, BY4741^COM^ *WHI2* was mostly resistant to hydrogen peroxide (only a 5% growth reduction), whereas BY4741^COM^ *WHI2*^G1324T^ was sensitive (40% growth reduction, Figure 4B). Deleting *YPK9* from BY4741^COM^ *WHI2*^G1324T^ rendered the strain very sensitive to hydrogen peroxide (61% growth reduction) and revealed a negative genetic interaction (ε = −0.24, *p*-value < 0.0001) between *ypk9*∆ and *WHI2*^G1324T^ under these conditions.

We next examined how the changes in *YPK9* and *WHI2* affect the hydrogen peroxide sensitivity of BY4742, the α mating type. BY4742, which has full-length *WHI2*, was not sensitive to hydrogen peroxide (Figure 4C). However, introducing *WHI2*’s premature stop codon into BY4742 yielded a strain (BY4742 *WHI2*^G1324T^) that was very sensitive to hydrogen peroxide (Figure 4C, 60% growth reduction). We deleted *YPK9* from BY4742 *WHI2* and showed that the resulting strain (BY4742 *ypk9*∆ *WHI2*) was not sensitive to hydrogen peroxide (Figure 4C). The combination of *WHI2*^G1324T^ and *ypk9*∆ in BY4742 was very sensitive to hydrogen peroxide but indistinguishable from that of BY4742 *WHI2*^G1324T^ (Figure 4C, 54% and 60% growth reduction, respectively). Thus, loss of *YPK9* from BY4742 *WHI2* provided no additional sensitivity to hydrogen peroxide. Together, these results support the idea that *WHI2* is a key regulator of oxidative stress in *S. cerevisiae* [27,28]. *YPK9*’s role in regulating oxidative stress is revealed in BY4741^COM^ but obscured in BY4742.

To determine if the WHI2^G1324T^ mutation is common in the yeast deletion collection or is unique to the commercial BY4741 isolate, we sequenced the *WHI2* gene in 20 randomly chosen strains from the yeast BY4741 deletion collection. All 20 strains contained full-length *WHI2*, indicating that the parental strain lacked the *WHI2*^G1324T^ mutation (Figure S1).

The differences in the genetic backgrounds of BY4741^COM^ and BY4741^YKO^, especially the SNP in *WHI2* (Table 1 and Figure 2), prompted us to sequence *WHI2* in several *S. cerevisiae* wildtype strains and compare them to previously reported *WHI2* sequences (Figure S2). The genomes of S288C, BY4741 (Stanford), BY4741 (Toronto), BY4741 (Euroscarf), and BY4742 (Euroscarf) encode full-length Whi2p. The genomes of BY4741 (Open Biosystems), BY4741^COM^ (GE Healthcare, Figure 2), and BY4741^COM^ *ypk9*∆ (this study, Figure S2) encode a truncated Whi2p of 441 amino acid residues. The genome of BY4742 (Toronto) encodes a truncated Whi2p of 369 amino acid residues, whereas that of BY4742 (Stanford) encodes a truncated Whi2p of 423 amino acid residues. Thus, BY4741 and BY4742 isolates have, respectively, at least two and three alleles of *WHI2*. The *WHI2* alleles can cause dramatic phenotypes, for example, the sensitivity to oxidative stress reported here. The different *WHI2* alleles found in commonly used *S. cerevisiae* wildtype isolates, including the **a** (BY4741) and α (BY4742) mating types, underscore the need for genomic sequence verification of wildtype isolates and heightened awareness within the yeast community.

## 4. Discussion

We showed above that much of the hydrogen peroxide sensitivity of BY4741^COM^ can be attributed to a G-to-T SNP at position 412,193 chr XV (*WHI2*) yielding a 441 amino acid-truncated version of Whi2p (Whi2p^E442*^). To the best of our knowledge, this truncation has not previously been reported. Strains lacking *YPK9* but containing full-length *WHI2* in the BY4741 background were only modestly sensitive to hydrogen peroxide (~10% growth reduction).

The transcription factors Msn2 and Msn4 were identified as master regulators of the general stress response, an umbrella term that includes nutrient starvation, heat shock, and oxidative stress [30]. Kaida et al. [27] showed that Psr1p/Psr2p phosphatases, Msn2p, and Whi2p physically interact and can be found together at the cell membrane. Upon oxidative stress, Msn2p is released from Psr1p and Whi2p. Msn2p enters the nucleus, where it activates transcription of stress response genes including catalase and superoxide dismutase. Whi2p binds Psr1p, and both are required for full activation of Msn2p [27]. Our results are consistent with the hypothesis that the C-terminal 45-amino-acid residues of Whi2p mediate this important role in regulating Msn2p activation during the oxidative stress response.

The putative parental wildtype strain BY4741^COM^ used in our studies has *WHI2*^G1324T^, whereas all twenty of the YKO collection BY4741 strains we sampled encoded full-length Whi2p. Thus, this BY4741^COM^ isolate is not isogenic with the parental strain used to construct the deletion collection. The genomes of the Stanford, Toronto, and Euroscarf BY4741 isolates encode full-length Whi2p, whereas that of Open Biosystem encodes the truncated Whi2p of 441 amino acid residues. In the case of BY4742, the genomes of the Toronto and Stanford isolates encode, respectively, the truncated Whi2p of 369- and 423-amino-acid residues. The genomes of the BY4742 isolate used in our studies and that of Euroscarf’s encode full-length Whi2p. The presence of variants in established wildtype strains and their potentially profound effect on phenotype, for example, the oxidative stress reported here, underscore the need for whole-genome sequencing of commonly used strains [31]. Not only will this resolve the interpretation of conflicting results, but also reveal the biological significance of fortuitous variants.

Other groups have reported informative mutations in *WHI2* [20,32,33]. About 30% of the strains sequenced during the construction of a global suppressor network contained at least one mutation in a group of six genes, one of which was *WHI2* [20]. Comyn et al. [32] identified *WHI2* secondary mutations in 13 strains from the deletion collection during a screen for genes important for protein quality control. The mutant strains were defective in degrading misfolded proteins resulting, perhaps, from disruption of the interaction between Whi2p and Psr1p/Msn2p and its effect on downstream factors [32]. Of the 13 strains, 11 had a premature stop codon in *WHI2*. The stop codons were evenly distributed throughout *WHI2* sequence. Cheng et al. [33] reported truncated versions of Whi2p of 27, 68, and 152 amino acid residues in length. Thus, *WHI2* is a member of a small group of genes for whom loss-of-function, or partial truncation, mutations appear to be frequently advantageous.

Although neither a genetic nor physical interaction between *YPK9* and *WHI2* has been previously reported, a comparison of their respective known interactors converges on eIF5A (Hyp2p). In *S. cerevisiae*, polyamines scavenge reactive oxygen species (ROS) and play a critical role in hypusination [34], a post-translational modification required for the activity of the essential eIF5A (Hyp2p). Whi2p physically interacts with Hyp2p, and *YPK9* and *HYP2* have a negative genetic interaction based on whole-gene knockout data [10,35]. Thus, Whi2p may block translation [9], in part through its interaction with eIF5A (Hyp2p). We showed a negative genetic interaction between *YPK9* and *WHI2* in the BY4741^COM^ background in the presence of hydrogen peroxide. When *S. cerevisiae* is treated with hydrogen peroxide, Tpo1p exports spermine and spermidine, thereby lowering their cytosolic concentrations [36]. Thus, it is tempting to speculate that in the presence of hydrogen peroxide, cell survival and hypusination are more dependent on Ypk9p to deliver polyamines to the cytosol. Although members of the mammalian *ATP13A3-5* family cannot suppress the Mn^2+^ toxicity of *ypk9*∆ strains [37], whether they complement growth defects during oxidative stress in these strains warrants further investigation.

In *S. cerevisiae*, cytosolic polyamines, which scavenge ROS, are derived from at least three routes: biosynthesis from ornithine [5], transport into the cell via polyamine transporters (e.g., Tpo1p [36]), and putative export from the vacuole via Ypk9p. The ability of *S. cerevisiae* to obtain polyamines from the three routes may explain the modest growth reduction of *ypk9*∆ strains in the presence of hydrogen peroxide, and this genetic redundancy may also reflect the necessity of controlling the cytosolic concentration of polyamines. The ability of spermidine to enhance longevity in yeast, flies, worms, human cells, and mice by inducing autophagy [38]; the interest in spermidine’s effect on cognition [39]; and the link between the polyamine pathway and Parkinson’s disease [40] underscore the need to further investigate polyamines and their regulators, such as *YPK9* and *WHI2*.

This fortuitous discovery of a negative, condition-dependent genetic interaction highlights the importance of multicondition mapping of genetic interactions in multiple backgrounds. While high-throughput reverse-genetics methods are becoming more precise at delivering particular modifications across the genome, unbiased forward genetics is still an important strategy for generating and dissecting unanticipated modes and complexity of genetic variation.

## Figures and Tables

**Table 1:** SNPs within ORFs determined by whole-genome sequencing.

					Strains			
Standard Name	Name Description	Chromosome Location	SNP	Amino Acid Change	BY4741^COM^	BY4741^COM^ *ypk9*∆	BY4741^YKO^ *ypk9*∆	BY4742
*ADH7*	Alcohol Dehydrogenase	III/ 309,566	G to A	Gly166Asp	+	+	−	−
*DNF2*	Drs2 Neo1 Family	IV/ 632,116	G to C	Gly279Arg	+	+	−	−
*WHI2*	Whiskey 2	XV/ 412,193	G to T	Glu442 *	+	+	−	−
*RAX1*	Revert to AXial 1	XV/ 881,528	C to A	Cys188 *	+	+	−	−

## Data Availability

The data presented in this study are available in the article and supplementary material.

## Supplementary Materials

The following are available online at https://www.mdpi.com/article/10.3390/microorganisms9122584/s1, Figure S1. Chromatograms of *WHI2* sequencing reactions; Figure S2. Whi2p amino acid sequence from select wildtype *S. cerevisiae* strains and isolates; Table S1. List of strains; Table S2. Summary of variants in (A) BY4741^COM^, (B) BY4741^COM^ *ypk9*∆, (C) BY4741^YKO^ *ypk9*∆, and (D) BY4742.

## References

[1] Gitler A.D., Chesi A., Geddie M.L., Strathearn K.E., Hamamichi S., Hill K.J., Caldwell K.A., Caldwell G.A., Cooper A.A., Rochet J.-C. (2009). Alpha-Synuclein is part of a diverse and highly conserved interaction network that includes PARK9 and manganese toxicity. Nat. Genet..

[2] Schmidt K., Wolfe D.M., Stiller B., Pearce D.A. (2009). Cd^2+^, Mn^2+^, Ni^2+^ and Se^2+^ toxicity to *Saccharomyces cerevisiae* lacking YPK9p the orthologue of human ATP13A2. Biochem. Biophys. Res. Commun..

[3] van Veen S., Martin S., Van den Haute C., Benoy V., Lyons J., Vanhoutte R., Kahler J.P., Decuypere J.-P., Gelders G., Lambie E. (2020). ATP13A2 deficiency disrupts lysosomal polyamine export. Nature.

[4] Ramirez A., Heimbach A., Gründemann J., Stiller B., Hampshire D., Cid L.P., Goebel I., Mubaidin A.F., Wriekat A.-L., Roeper J. (2006). Hereditary parkinsonism with dementia is caused by mutations in ATP13A2, encoding a lysosomal type 5 P-type ATPase. Nat. Genet..

[5] Tabor C.W., Tabor H. (1985). Polyamines in microorganisms. Microbiol. Rev..

[6] Pegg A.E. (2009). Mammalian polyamine metabolism and function. IUBMB Life.

[7] Sudbery P.E., Goodey A.R., Carter B.L.A. (1980). Genes which control cell proliferation in the yeast *Saccharomyces cerevisiae*. Nature.

[8] Teng X., Yau E., Sing C., Hardwick J.M. (2018). Whi2 signals low leucine availability to halt yeast growth and cell death. FEMS Yeast Res..

[9] Chen X., Wang G., Zhang Y., Dayhoff-Brannigan M., Diny N.L., Zhao M., He G., Sing C.N., Metz K.A., Stolp Z.D. (2018). Whi2 is a conserved negative regulator of TORC1 in response to low amino acids. PLoS Genet..

[10] Costanzo M., Baryshnikova A., Bellay J., Kim Y., Spear E.D., Sevier C.S., Ding H., Koh J.L., Toufighi K., Mostafavi S. (2010). The Genetic Landscape of a Cell. Science.

[11] Engel S.R., Dietrich F.S., Fisk D.G., Binkley G., Balakrishnan R., Costanzo M.C., Dwight S.S., Hitz B.C., Karra K., Nash R.S. (2014). The Reference Genome Sequence of *Saccharomyces cerevisiae*: Then and Now. G3: Genes Genomes Genet..

[12] Li H., Durbin R. (2009). Fast and accurate short read alignment with Burrows-Wheeler transform. Bioinformatics.

[13] Gietz R.D., Schiestl R.H. (2007). High-efficiency yeast transformation using the LiAc/SS carrier DNA/PEG method. Nat. Protoc..

[14] Goldstein A.L., McCusker J.H. (1999). Three new dominant drug resistance cassettes for gene disruption in *Saccharomyces cerevisiae*. Yeast.

[15] Hoffman C.S., Winston F. (1987). A ten-minute DNA preparation from yeast efficiently releases autonomous plasmids for transformaion of *Escherichia coli*. Gene.

[16] Giaever G., Chu A.M., Ni L., Connelly C., Riles L., Véronneau S., Dow S., Lucau-Danila A., Anderson K., André B. (2002). Functional profiling of the *Saccharomyces cerevisiae* genome. Nature.

[17] Koh J.L.Y., Ding H., Costanzo M., Baryshnikova A., Toufighi K., Bader G., Myers C.L., Andrews B.J., Boone C. (2009). DRYGIN: A database of quantitative genetic interaction networks in yeast. Nucleic Acids Res..

[18] Teng X., Hardwick J.M. (2013). Quantification of Genetically Controlled Cell Death in Budding Yeast. Springer Protoc. Handb..

[19] Larroy C., Pares X., Biosca J.A. (2002). Characterization of a *Saccharomyces cerevisiae* NADP(H)-dependent alcohol dehydrogenase (ADHVII), a member of the cinnamyl alcohol dehydrogenase family. Eur. J. Biochem..

[20] van Leeuwen J., Pons C., Mellor J.C., Yamaguchi T.N., Friesen H., Koschwanez J., Ušaj M.M., Pechlaner M., Takar M., Ušaj M. (2016). Exploring genetic suppression interactions on a global scale. Science.

[21] Hua Z., Fatheddin P., Graham T.R. (2002). An Essential Subfamily of Drs2p-related P-Type ATPases Is Required for Protein Trafficking between Golgi Complex and Endosomal/Vacuolar System. Mol. Biol. Cell.

[22] Pomorski T.G., Lombardi R., Riezman H., Devaux P.F., van Meer G., Holthuis J.C.M. (2003). Drs2p-related P-type ATPases Dnf1p and Dnf2p Are Required for Phospholipid Translocation across the Yeast Plasma Membrane and Serve a Role in Endocytosis. Mol. Biol. Cell.

[23] Cherry J.M., Hong E.L., Amundsen C., Balakrishnan R., Binkley G., Chan E., Christie K., Costanzo M., Dwight S.S., Engel S. (2011). Saccharomyces Genome Database: The genomics resource of budding yeast. Nucleic Acids Res..

[24] Chen T., Hiroko T., Chaudhuri A., Inose F., Lord M., Tanaka S., Chant J., Fujita A. (2000). Multigenerational Cortical Inheritance of the Rax2 Protein in Orienting Polarity and Division in Yeast. Science.

[25] Fujita A., Lord M., Hiroko T., Hiroko F., Chen T., Oka C., Misumi Y., Chant J. (2004). Rax1, a protein required for the establishment of the bipolar budding pattern in yeast. Gene.

[26] Teng X., Hardwick J.M. (2019). Whi2: A new player in amino acid sensing. Curr. Genet..

[27] Kaida D., Yashiroda H., Toh-e A., Kikuchi Y. (2002). Yeast Whi2 and Psr1-phosphatase form a complex and regulate STRE-mediated gene expression. Genes Cells.

[28] Sadeh A., Movshovich N., Volokh M., Gheber L., Aharoni A. (2011). Fine-tuning of the Msn2/4–mediated yeast stress responses as revealed by systematic deletion of Msn2/4 partners. Mol. Biol. Cell.

[29] Sievers F., Wilm A., Dineen D., Gibson T.J., Karplus K., Li W., López R., McWilliam H., Remmert M., Söding J. (2011). Fast, scalable generation of high-quality protein multiple sequence alignments using Clustal Omega. Mol. Syst. Biol..

[30] Martinez-Pastor M.T., Marchler G., Schuller C., Marchler-Bauer A., Ruis H., Estruch F. (1996). The *Saccharomyces cerevisiae* zinc finger proteins Msn2p and Msn4p are required for transcriptional induction through the stress response element (STRE). EMBO J..

[31] Gallegos J.E., Hayrynen S., Adames N.R., Peccoud J. (2020). Challenges and opportunities for strain verification by whole-genome sequencing. Sci. Rep..

[32] Comyn S.A., Flibotte S., Mayor T. (2017). Recurrent background mutations in WHI2 impair proteostasis and degradation of misfolded cytosolic proteins in *Saccharomyces cerevisiae*. Sci. Rep..

[33] Cheng W.-C., Teng X., Park H.K., Tucker C.M., Dunham M., Hardwick J.M. (2008). Fis1 deficiency selects for compensatory mutations responsible for cell death and growth control defects. Cell Death Differ..

[34] Pegg A.E. (2016). Functions of Polyamines in Mammals. J. Biol. Chem..

[35] Ho Y., Gruhler A., Heilbut A., Bader G.D., Moore L., Adams S.-L., Millar A., Taylor P., Bennett K.L., Boutilier K. (2002). Systematic identification of protein complexes in *Saccharomyces cerevisiae* by mass spectrometry. Nature.

[36] Krüger A., Vowinckel J., Mülleder M., Grote P., Capuano F., Bluemlein K., Ralser M. (2013). Tpo1-mediated spermine and spermidine export controls cell cycle delay and times antioxidant protein expression during the oxidative stress response. EMBO Rep..

[37] Sørensen D.M., Holemans T., Van Veen S., Martin S., Arslan T., Haagendahl I.W., Holen H.W., Hamouda N.N., Eggermont J., Palmgren M. (2018). Parkinson disease related ATP13A2 evolved early in animal evolution. PLoS ONE.

[38] Eisenberg T., Knauer H., Schauer A., Büttner S., Ruckenstuhl C., Carmona-Gutierrez D., Ring J., Schroeder S., Magnes C., Antonacci L. (2009). Induction of autophagy by spermidine promotes longevity. Nat. Cell Biol..

[39] Wirth M., Schwarz C., Benson G., Horn N., Buchert R., Lange C., Köbe T., Hetzer S., Maglione M., Michael E. (2019). Effects of spermidine supplementation on cognition and biomarkers in older adults with subjective cognitive decline (SmartAge)—study protocol for a randomized controlled trial. Alzheimer’s Res. Ther..

[40] Lewandowski N.M., Ju S., Verbitsky M., Ross B., Geddie M.L., Rockenstein E., Adame A., Muhammad A., Vonsattel J.P., Ringe D. (2010). Polyamine pathway contributes to the pathogenesis of Parkinson disease. Proc. Natl. Acad. Sci. USA.

