# Supplementary material for: YPK9 and WHI2 Negatively Interact during Oxidative Stress
Source: Microorganisms. 2021 Dec 14;9(12):2584. doi: 10.3390/microorganisms9122584 (PMC8705791; doi:10.3390/microorganisms9122584)
Supplement: Supplementary file 1 [file microorganisms-09-02584-s001.zip › microorganisms-1447616-supplementary.pdf]

microorganisms

Supplementary Materials

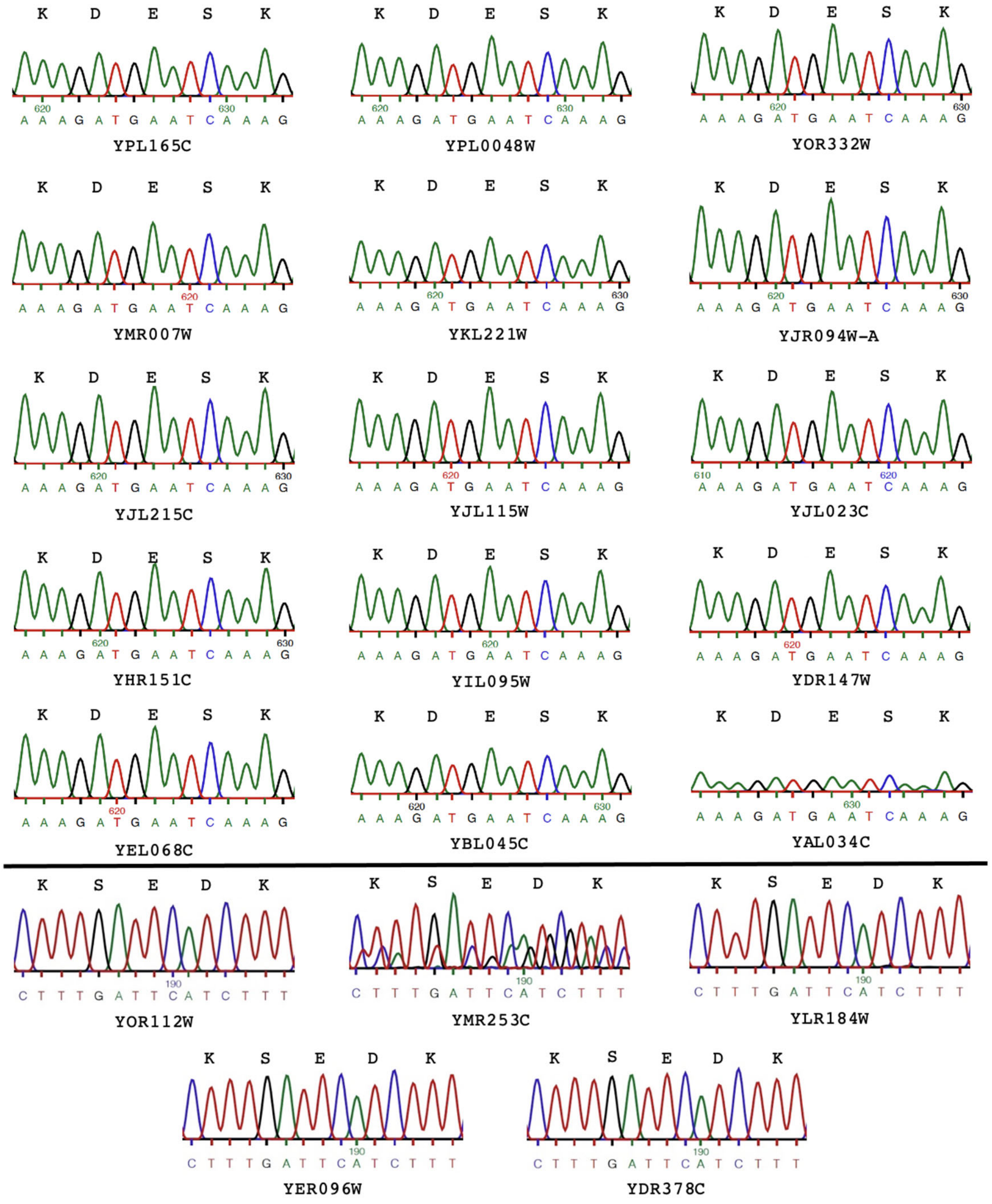

Figure S1. Chromatograms of *WHI2* sequencing reactions. Genomic DNA was isolated from twenty randomly chosen strains from the BY4741 deletion collection and *WHI2* was sequenced. The sequence of Whi2p (amino acids 440 - 444) and the systematic name for each deleted gene are shown above and below the chromatograms, respectively. Sequences above the line were obtained using a *WHI2* forward primer, whereas those below were obtained using the reverse primer. None of the strains had the BY4741^{COM} premature stop codon yielding Whi2p^{E442*}.

```
S288C                     MDDIITQVSPDNAESAPILQEQQQQQNSQYEGNEEDYGDSLIHLNIQENHYFITRDQLMS 60
BY4741_OpenBiosystems     MDDIITQVSPDNAESAPILQEQQQQQNSQYEGNEEDYGDSLIHLNIQENHYFITRDQLMS 60
BY4741_COM_Δypk9          MDDIITQVSPDNAESAPILQEQQQQQNSQYEGNEEDYGDSLIHLNIQENHYFITRDQLMS 60
BY4741_Stanford           MDDIITQVSPDNAESAPILQEQQQQQNSQYEGNEEDYGDSLIHLNIQENHYFITRDQLMS 60
BY4741_Toronto            MDDIITQVSPDNAESAPILQEQQQQQNSQYEGNEEDYGDSLIHLNIQENHYFITRDQLMS 60
BY4741_Euroscarf          MDDIITQVSPDNAESAPILQEQQQQQNSQYEGNEEDYGDSLIHLNIQENHYFITRDQLMS 60
BY4742_Stanford           MDDIITQVSPDNAESAPILQEQQQQQNSQYEGNEEDYGDSLIHLNIQENHYFITRDQLMS 60
BY4742_Toronto            MDDIITQVSPDNAESAPILQEQQQQQNSQYEGNEEDYGDSLIHLNIQENHYFITRDQLMS 60
BY4742_Euroscarf          MDDIITQVSPDNAESAPILQEQQQQQNSQYEGNEEDYGDSLIHLNIQENHYFITRDQLMS 60
                          ************************************************************

S288C                     LPESLLLCLFPSGVFLDRCGQVITNLTRDDEVYIVNFPPDCFEYIMEIYTKAHDDLYNHP 120
BY4741_OpenBiosystems     LPESLLLCLFPSGVFLDRCGQVITNLTRDDEVYIVNFPPDCFEYIMEIYTKAHDDLYNHP 120
BY4741_COM_Δypk9          LPESLLLCLFPSGVFLDRCGQVITNLTRDDEVYIVNFPPDCFEYIMEIYTKAHDDLYNHP 120
BY4741_Stanford           LPESLLLCLFPSGVFLDRCGQVITNLTRDDEVYIVNFPPDCFEYIMEIYTKAHDDLYNHP 120
BY4741_Toronto            LPESLLLCLFPSGVFLDRCGQVITNLTRDDEVYIVNFPPDCFEYIMEIYTKAHDDLYNHP 120
BY4741_Euroscarf          LPESLLLCLFPSGVFLDRCGQVITNLTRDDEVYIVNFPPDCFEYIMEIYTKAHDDLYNHP 120
BY4742_Stanford           LPESLLLCLFPSGVFLDRCGQVITNLTRDDEVYIVNFPPDCFEYIMEIYTKAHDDLYNHP 120
BY4742_Toronto            LPESLLLCLFPSGVFLDRCGQVITNLTRDDEVYIVNFPPDCFEYIMEIYTKAHDDLYNHP 120
BY4742_Euroscarf          LPESLLLCLFPSGVFLDRCGQVITNLTRDDEVYIVNFPPDCFEYIMEIYTKAHDDLYNHP 120
                          ************************************************************

S288C                     VEKFFDRPSSSFVSNAKGFFGLSSNNSISSNNEQDILHQKPAIIVLREDLDYYCVPQEEF 180
BY4741_OpenBiosystems     VEKFFDRPSSSFVSNAKGFFGLSSNNSISSNNEQDILHQKPAIIVLREDLDYYCVPQEEF 180
BY4741_COM_Δypk9          VEKFFDRPSSSFVSNAKGFFGLSSNNSISSNNEQDILHQKPAIIVLREDLDYYCVPQEEF 180
BY4741_Stanford           VEKFFDRPSSSFVSNAKGFFGLSSNNSISSNNEQDILHQKPAIIVLREDLDYYCVPQEEF 180
BY4741_Toronto            VEKFFDRPSSSFVSNAKGFFGLSSNNSISSNNEQDILHQKPAIIVLREDLDYYCVPQEEF 180
BY4741_Euroscarf          VEKFFDRPSSSFVSNAKGFFGLSSNNSISSNNEQDILHQKPAIIVLREDLDYYCVPQEEF 180
BY4742_Stanford           VEKFFDRPSSSFVSNAKGFFGLSSNNSISSNNEQDILHQKPAIIVLREDLDYYCVPQEEF 180
BY4742_Toronto            VEKFFDRPSSSFVSNAKGFFGLSSNNSISSNNEQDILHQKPAIIVLREDLDYYCVPQEEF 180
BY4742_Euroscarf          VEKFFDRPSSSFVSNAKGFFGLSSNNSISSNNEQDILHQKPAIIVLREDLDYYCVPQEEF 180
                          ************************************************************

S288C                     QFDSTNEENNEDLLRHFMAQVKMAAGSYLTSKTSIFQGLYSSNRLKQQQQQQKIEKGSNS 240
BY4741_OpenBiosystems     QFDSTNEENNEDLLRHFMAQVKMAAGSYLTSKTSIFQGLYSSNRLKQQQQQQKIEKGSNS 240
BY4741_COM_Δypk9          QFDSTNEENNEDLLRHFMAQVKMAAGSYLTSKTSIFQGLYSSNRLKQQQQQQKIEKGSNS 240
BY4741_Stanford           QFDSTNEENNEDLLRHFMAQVKMAAGSYLTSKTSIFQGLYSSNRLKQQQQQQKIEKGSNS 240
BY4741_Toronto            QFDSTNEENNEDLLRHFMAQVKMAAGSYLTSKTSIFQGLYSSNRLKQQQQQQKIEKGSNS 240
BY4741_Euroscarf          QFDSTNEENNEDLLRHFMAQVKMAAGSYLTSKTSIFQGLYSSNRLKQQQQQQKIEKGSNS 240
BY4742_Stanford           QFDSTNEENNEDLLRHFMAQVKMAAGSYLTSKTSIFQGLYSSNRLKQQQQQQKIEKGSNS 240
BY4742_Toronto            QFDSTNEENNEDLLRHFMAQVKMAAGSYLTSKTSIFQGLYSSNRLKQQQQQQKIEKGSNS 240
BY4742_Euroscarf          QFDSTNEENNEDLLRHFMAQVKMAAGSYLTSKTSIFQGLYSSNRLKQQQQQQKIEKGSNS 240
                          ************************************************************

S288C                     SSNTKSTSKKLGPAEQHLMDMLCSSGFTKETCWGNRTQETGKTVISSLSLCRLANETTEG 300
BY4741_OpenBiosystems     SSNTKSTSKKLGPAEQHLMDMLCSSGFTKETCWGNRTQETGKTVISSLSLCRLANETTEG 300
BY4741_COM_Δypk9          SSNTKSTSKKLGPAEQHLMDMLCSSGFTKETCWGNRTQETGKTVISSLSLCRLANETTEG 300
BY4741_Stanford           SSNTKSTSKKLGPAEQHLMDMLCSSGFTKETCWGNRTQETGKTVISSLSLCRLANETTEG 300
BY4741_Toronto            SSNTKSTSKKLGPAEQHLMDMLCSSGFTKETCWGNRTQETGKTVISSLSLCRLANETTEG 300
BY4741_Euroscarf          SSNTKSTSKKLGPAEQHLMDMLCSSGFTKETCWGNRTQETGKTVISSLSLCRLANETTEG 300
BY4742_Stanford           SSNTKSTSKKLGPAEQHLMDMLCSSGFTKETCWGNRTQETGKTVISSLSLCRLANETTEG 300
BY4742_Toronto            SSNTKSTSKKLGPAEQHLMDMLCSSGFTKETCWGNRTQETGKTVISSLSLCRLANETTEG 300
BY4742_Euroscarf          SSNTKSTSKKLGPAEQHLMDMLCSSGFTKETCWGNRTQETGKTVISSLSLCRLANETTEG 300
                          ************************************************************

S288C                     FRQKFNEAKAKWEAEHKPSQDNFITPMQSNISINSLSASKSNSTISTARNLTSGSTAPAT 360
BY4741_OpenBiosystems     FRQKFNEAKAKWEAEHKPSQDNFITPMQSNISINSLSASKSNSTISTARNLTSGSTAPAT 360
BY4741_COM_Δypk9          FRQKFNEAKAKWEAEHKPSQDNFITPMQSNISINSLSASKSNSTISTARNLTSGSTAPAT 360
BY4741_Stanford           FRQKFNEAKAKWEAEHKPSQDNFITPMQSNISINSLSASKSNSTISTARNLTSGSTAPAT 360
BY4741_Toronto            FRQKFNEAKAKWEAEHKPSQDNFITPMQSNISINSLSASKSNSTISTARNLTSGSTAPAT 360
BY4741_Euroscarf          FRQKFNEAKAKWEAEHKPSQDNFITPMQSNISINSLSASKSNSTISTARNLTSGSTAPAT 360
BY4742_Stanford           FRQKFNEAKAKWEAEHKPSQDNFITPMQSNISINSLSASKSNSTISTARNLTSGSTAPAT 360
BY4742_Toronto            FRQKFNEAKAKWEAEHKPSQDNFITPMQSNISINSLSASKSNSTISTARNLTSGSTAPAT 360
BY4742_Euroscarf          FRQKFNEAKAKWEAEHKPSQDNFITPMQSNISINSLSASKSNSTISTARNLTSGSTAPAT 360
                          ************************************************************

S288C                     ARDKRKSRLSKLADNVRSHSSSRHSSQTRSKPPELPKLYDLVPKPNINAKLLLFWRKPAR 420
BY4741_OpenBiosystems     ARDKRKSRLSKLADNVRSHSSSRHSSQTRSKPPELPKLYDLVPKPNINAKLLLFWRKPAR 420
BY4741_COM_Δypk9          ARDKRKSRLSKLADNVRSHSSSRHSSQTRSKPPELPKLYDLVPKPNINAKLLLFWRKPAR 420
BY4741_Stanford           ARDKRKSRLSKLADNVRSHSSSRHSSQTRSKPPELPKLYDLVPKPNINAKLLLFWRKPAR 420
BY4741_Toronto            ARDKRKSRLSKLADNVRSHSSSRHSSQTRSKPPELPKLYDLVPKPNINAKLLLFWRKPAR 420
BY4741_Euroscarf          ARDKRKSRLSKLADNVRSHSSSRHSSQTRSKPPELPKLYDLVPKPNINAKLLLFWRKPAR 420
BY4742_Stanford           ARDKRKSRLSKLADNVRSHSSSRHSSQTRSKPPELPKLYDLVPKPNINAKLLLFWRKPAR 420
BY4742_Toronto            ARDKRKSRL--------------------------------------------------- 369
BY4742_Euroscarf          ARDKRKSRLSKLADNVRSHSSSRHSSQTRSKPPELPKLYDLVPKPNINAKLLLFWRKPAR 420
                          *********

S288C                     KCWWGEEDIELEVEVFGSWKDESKKIIELILPTNVDPEAELHKIIVPVRLHIRRVWTLEL 480
BY4741_OpenBiosystems     KCWWGEEDIELEVEVFGSWKD--------------------------------------- 441
BY4741_COM_Δypk9          KCWWGEEDIELEVEVFGSWKD--------------------------------------- 441
BY4741_Stanford           KCWWGEEDIELEVEVFGSWKDESKKIIELILPTNVDPEAELHKIIVPVRLHIRRVWTLEL 480
BY4741_Toronto            KCWWGEEDIELEVEVFGSWKDESKKIIELILPTNVDPEAELHKIIVPVRLHIRRVWTLEL 480
BY4741_Euroscarf          KCWWGEEDIELEVEVFGSWKDESKKIIELILPTNVDPEAELHKIIVPVRLHIRRVWTLEL 480
BY4742_Stanford           KCW--------------------------------------------------------- 423
BY4742_Toronto            ------------------------------------------------------------ 369
BY4742_Euroscarf          KCWWGEEDIELEVEVFGSWKDESKKIIELILPTNVDPEAELHKIIVPVRLHIRRVWTLEL 480


S288C                     SVIGVQ      486
BY4741_OpenBiosystems     ------      441
BY4741_COM_Δypk9          ------      441
BY4741_Stanford           SVIGVQ      486
BY4741_Toronto            SVIGVQ      486
BY4741_Euroscarf          SVIGVQ      486
BY4742_Stanford           ------      423
BY4742_Toronto            ------      369
BY4742_Euroscarf          SVIGVQ      486
```

Figure S2. Whi2p amino acid sequence from select wildtype *S. cerevisiae* strains and isolates. The Whi2p sequences from S288C, Stanford (BY4741 and BY4742), and Toronto (BY4741 and BY4742) were obtained from the SGD. We sequenced *WHI2* from Open Biosystems (BY4741), BY4741^{COM} *ypk9*Δ, and Euroscarf (BY4741 and BY4742).

Table S1. List of strains.

Strain Name	Genotype	Source
S288C	*MATα SUC2 gal2 mal2 mel flo1 flo8-1 hap1 ho bio1 bio6*	ATCC
BY4741^{COM}	*MAT**a** his3Δ1 leu2Δ0 met15Δ0 ura3Δ0 WHI2*^{G1324T}	GE Healthcare
BY4741	*MAT**a** his3Δ1 leu2Δ0 met15Δ0 ura3Δ0 WHI2*^{G1324T}	Open Biosystems
BY4741	*MATα his3Δ1 leu2Δ0 lys2Δ0 ura3Δ0 WHI2*	Euroscarf
BY4742	*MATα his3Δ1 leu2Δ0 lys2Δ0 ura3Δ0 WHI2*	Gift from David Gresham
BY4742	*MATα his3Δ1 leu2Δ0 lys2Δ0 ura3Δ0 WHI2*	Euroscarf
YKO *ypk9Δ*	BY4741, *ypk9Δ*::KAN *WHI2*	Thermo Scientific
1919	BY4742, *WHI2*::*WHI2*^{G1324T}-Hyg	This study
1920	BY4741^{COM}, *WHI2*^{G1324T}::*WHI2*-Hyg	This study
1923	BY4742, *ypk9Δ*::NTC *WHI2*::*WHI2*^{G1324T}-Hyg	This study
1924	YKO *ypk9Δ*, *WHI2*::*WHI2*^{G1324T}-Hyg	This study
1926	BY4742, *ypk9Δ*::NTC	This study
1927	BY4741^{COM}, *ypk9Δ*::KAN *WHI2*^{G1324T}	This study

Table S2. Summary of variants in (A) BY4741^{COM} , (B) BY4741^{COM} *ypk9Δ*, (C) BY4741^{YKO} *ypk9Δ*, and (D) BY4742.

Chromosome	A	B	C	D	A/B	A/C	A/D	A/B/D	A/B/C	A/C/D	B/C	B/D	B/C/D	C/D	ABCD	TOTAL
I	2	2	2	2	0	3	1	1	0	0	0	0	0	0	48	61
II	0	1	0	5	1	0	0	0	0	1	0	0	0	0	23	31
III	0	0	0	1	2	0	0	0	1	0	0	1	0	0	10	15
IV	0	0	0	2	1	0	1	0	0	0	1	0	1	0	27	33
V	0	0	1	0	0	0	0	0	0	0	0	0	0	0	4	5
VI	0	0	0	1	0	0	0	0	0	0	0	0	0	0	2	3
VII	0	0	1	0	0	0	0	0	1	0	0	0	0	1	11	14
VIII	0	1	0	1	1	0	0	0	1	0	0	1	0	0	4	9
IX	1	1	0	0	0	0	0	0	0	1	0	0	0	0	5	8
X	0	4	0	0	0	0	0	0	1	0	1	0	0	0	11	17
XI	0	1	0	1	0	0	0	0	1	0	0	0	0	0	2	5
XII	0	1	5	1	0	0	0	0	2	0	0	0	0	0	11	20
XIII	1	0	0	8	0	0	0	0	1	1	0	0	0	0	16	27
XIV	0	1	0	1	2	0	0	0	0	0	0	1	0	0	8	13
XV	0	0	0	0	2	0	0	0	1	0	0	0	0	0	10	13
XVI	0	0	0	0	0	0	0	2	1	0	0	0	0	0	3	6
M	0	0	1	1	0	0	0	0	0	0	0	0	1	0	7	10
TOTAL	4	12	10	24	9	3	2	3	10	3	2	3	2	1	202	290
